# Vitamin D Receptor Gene Variants Associated with Serum 25(OH)D_3_ Levels in Patients with Dry Eye Syndrome

**DOI:** 10.3390/life15101552

**Published:** 2025-10-03

**Authors:** Borivoje Savic, Svetlana Stanojlovic, Bozidar Savic, Jelena Kostic, Margita Lucic, Katarina Jankovic Terzic, Bojana Dacic-Krnjaja

**Affiliations:** 1 University Clinical Centre of Serbia, University Eye Hospital, Pasterova 2, 11000 Belgrade, Serbia; 2 Faculty of Medicine, University of Belgrade, 11000 Belgrade, Serbia; 3 Institute of Veterinary Medicine of Serbia, Janisa Janulisa 14, 11000 Belgrade, Serbia; 4 Specialist Ophthalmology Clinic and Optical Shop OKOiOKO, Mekenzijeva 20, 11000 Belgrade, Serbia

**Keywords:** dry eye syndrome, vitamin D, VDR gene, polymorphisms, ApaI, personalized medicine, ocular immunology

## Abstract

Introduction: Dry Eye Syndrome (DES) is a multifactorial disorder of the ocular surface, characterized by complex interactions between environmental factors, immune dysregulation, and potential genetic predispositions. Vitamin D deficiency, known for its immunomodulatory properties, has increasingly been implicated in the pathogenesis of DES; however, the underlying mechanisms remain insufficiently elucidated. Of particular interest is the vitamin D receptor (VDR) gene, whose polymorphisms may influence the bioavailability and biological activity of vitamin D. Objective: The aim of this study was to investigate the association between serum 25-hydroxyvitamin D [25(OH)D_3_] levels and selected polymorphisms in the VDR gene (Taq, Fok, Apa, and Bsm) in patients with DES and to analyze their potential clinical and genetic interactions. Methods: This prospective observational study included 60 patients with a confirmed diagnosis of DES. Serum 25(OH)D_3_ levels were measured, and genotyping of four VDR single-nucleotide polymorphisms (SNPs) was performed using PCR followed by restriction fragment length polymorphism analysis. Genotype distributions were assessed in relation to vitamin D status using appropriate statistical tests and Hardy–Weinberg equilibrium analysis. Results: Over 85% of patients exhibited insufficient or deficient vitamin D levels. Among the analyzed SNPs, only the ApaI polymorphism (rs7975232) showed a statistically significant association with vitamin D status (*p* = 0.0384), with the homozygous AA genotype being more prevalent among patients with hypovitaminosis. The remaining polymorphisms (TaqI, FokI, BsmI) did not reach statistical significance; however, potential trends were observed that may warrant further investigation in larger cohorts. Conclusion: The findings suggest a potential role for VDR gene variability in the regulation of systemic vitamin D levels in patients with DES. Identification of specific genotypes may contribute to the development of personalized diagnostic and therapeutic strategies, particularly for patients with treatment-resistant forms of the disease. These results support the integration of genetic biomarkers and nutritional parameters into modern ophthalmologic practice.

## 1. Introduction

DES is a highly complex condition in which numerous environmental influences converge with genetic factors. Its global prevalence in the adult population ranges from 5% to more than 40%, depending on the cohort study [1]. As such, DES constitutes a significant public health challenge worldwide. Despite extensive clinical research, a definitive correlation between symptoms, clinical signs, and the underlying etiology has yet to be established. Multiple investigations have implicated diverse etiological determinants—including depression and anxiety [2,3,4], various hormonal alterations [5,6,7,8], specific pharmacotherapies [9], and neurological changes [10,11,12]—each contributing to DES to a greater or lesser extent. Although many lifestyle-related and comorbidity-associated risk factors have been identified, the overall pathogenic landscape remains incompletely defined, and the contribution of genetic factors, in particular, is still poorly understood. In addition to lifestyle-related and comorbidity-associated risk factors, increasing attention is being directed toward genetically determined predispositions that may contribute to the development of DES. Although the precise molecular mechanisms remain incompletely understood, emerging data from genetic studies provide a strong foundation for the hypothesis that genetic factors play a significant role in both the pathogenesis and clinical expression of DES. Numerous genetic investigations support this view. For example, twin studies have demonstrated a substantial heritability of phenotypic traits related to tear film parameters [13]. Furthermore, associations have been reported in patients with Sjögren’s syndrome and rheumatoid arthritis involving polymorphisms in genes such as TRIM21, STAT4, PTPN22, and MUC1 [14]. A large, recent genome-wide association study (GWAS) identified multiple loci associated with DES and revealed genetic links with several chronic conditions, including fibromyalgia and post-traumatic stress disorder (PTSD), suggesting a broader neuroimmunological and genetic correlation [15]. The gene encoding the vitamin D receptor (VDR) is located on human chromosome 12 (12q13.11) and comprises 11 exons. It encodes a nuclear hormone receptor for vitamin D_3_, belonging to the family of ligand-dependent transcriptional regulators, structurally related to steroid and thyroid hormone receptors [16]. The primary functions of the vitamin D receptor (VDR) involve the regulation of mineral metabolism; however, its activity extends beyond calcium and phosphate homeostasis, influencing a variety of other metabolic pathways, including those related to immune responses and carcinogenesis [17]. SNPs in the VDR gene have been implicated in the pathogenesis of systemic lupus erythematosus (SLE), a condition that often includes DES among its clinical manifestations [18,19]. Variants such as FokI (rs2228570), BsmI (rs1544410), ApaI (rs7975232), and TaqI (rs731236) have been extensively studied in the context of SLE and other autoimmune diseases [18,20]. These four SNPs were likewise investigated in our study, which focuses on DES patients with no history of ocular comorbidities, previous ocular surgeries, anemia, or systemic and neurological disorders. Our findings reinforce the concept of DES as a multifactorial disorder affecting both the tear film and ocular surface. The clinical manifestations of DES are heterogeneous, ranging from mild discomfort to marked visual instability, all of which significantly impair quality of life and, most importantly, reduce occupational productivity. Despite a steady increase in the global prevalence of DES, its underlying pathophysiological mechanisms remain only partially understood. In this context, the contribution of genetic determinants is gaining increasing attention within the field of clinical ophthalmology.

In addition to the clearly identified etiological factors discussed above, the role of chronic inflammation and immune dysregulation must be emphasized in the pathogenesis of DES as a distinct clinical entity. Dysfunction in tear film production initiates a local inflammatory response, which, over time, evolves into a chronic process characterized by increased secretion of proinflammatory interleukins—such as IL-1 and IL-6—and activation of T lymphocyte subsets, particularly CD4+ Th1 and Th17 cells. This immune cascade results in sustained inflammation that further damages the ocular surface epithelium, thereby compromising the tear film’s lipid and protein-based protective functions [21,22]. A strong correlation exists between systemic autoimmune diseases and DES. Autoantibodies such as anti-SSA/Ro and anti-SSB/La directly impair the function of the lacrimal glands. Patients with Sjögren’s syndrome, SLE, and rheumatoid arthritis represent well-documented populations in which DES is commonly observed, underscoring the immunopathological basis of the disease in these settings [23,24]. The diagnosis of DES remains a clinical challenge, requiring the integration of conventional diagnostic tools—such as the Schirmer test and the Ocular Surface Disease Index (OSDI)—with molecular biomarkers and clinical signs [25]. Modern advances in the detection and quantification of inflammatory mediators in tear fluid hold considerable potential to enhance diagnostic precision [26]. Therapeutic strategies are also evolving; while conventional treatment algorithms remain relevant, there is a growing shift toward personalized medicine tailored to the individual immunological and molecular profile of the patient. Genetics, in this context, represent a definitive biomarker and must be recognized as a major contributor to disease susceptibility and progression. Individualized treatment strategies are increasingly dependent on specific genetic predispositions and the patient’s unique immunological profile. Biologic therapies, by design, act through the modulation of genetically influenced inflammatory pathways. Furthermore, regenerative approaches—such as autologous serum eye drops—have demonstrated promising efficacy in severe and refractory cases, positioning them as a cornerstone of future therapeutic paradigms for dry eye disease [27,28]. We conclude that DES is far more than a localized ophthalmic disorder; rather, it reflects a complex condition situated at the intersection of systemic and neuroimmunological processes. Therefore, further integrated and multidisciplinary research is essential to elucidate the remaining pathophysiological questions—particularly those involving intricate gene–environment interactions. Such an approach would enable the development of more refined diagnostic models and more effective, personalized therapeutic strategies tailored to the specific biological and clinical needs of each patient.

## 2. Materials and Methods

This study was approved by the Ethics Committee of the University Clinical Center of Serbia in Belgrade (Decision No. 668/2-07, dated 29 September 2025). All participants received detailed information about the study and signed a written informed consent form in accordance with the principles of informed consent. This study was conducted in compliance with the Declaration of Helsinki. A total of sixty patients were enrolled in this prospective observational study. Participants were recruited from the Department of Ophthalmology at the University Clinical Center of Serbia in Belgrade and followed between January 2023 and December 2025. All patients were over 18 years of age, regardless of sex. The study sample included individuals diagnosed with DES who were under clinical observation at our institution during the specified period.

The diagnostic criteria for DES included the following: (1) the presence of one or more symptoms such as burning, irritation, foreign body sensation or grittiness, photophobia, ocular pain, dryness, or general discomfort; (2) a Schirmer test result of less than 10 mm/5 min in at least one eye, performed using Whatman filter paper strips; (3) positive staining of the cornea and/or conjunctiva with rose bengal, with a score greater than 1. Exclusion criteria included a history of ocular comorbidities, previous ocular surgeries, any form of anemia, and systemic and neurological disorders.

### 2.1. Measurement of Plasma 25(OH)D_3_ Concentration

Peripheral venous blood samples (5 mL) were collected for analysis of serum 25-hydroxyvitamin D_3_ [25(OH)D_3_] levels and for the detection of vitamin D receptor (VDR) gene polymorphisms. A 1 mL aliquot of each sample was used for DNA isolation, while the remaining portion was centrifuged to separate plasma for 25(OH)D_3_ quantification.

Serum 25(OH)D_3_ concentrations were measured using the Abbott 25OHD assay kit (Abbott Laboratories, North Chicago, IL, USA). The results were interpreted according to the following criteria: concentrations > 30 ng/mL were considered sufficient, values between 20 and 30 ng/mL were classified as insufficient, and levels < 20 ng/mL indicated vitamin D deficiency.

### 2.2. Genotyping of VDR Gene SNPs

Genomic DNA was extracted from peripheral blood leukocytes using the PureLink^®^ Genomic DNA Kit (K182001; Invitrogen, Carlsbad, CA, USA), following the manufacturer’s protocol [29]. Extracted DNA samples were stored at −80 °C until analysis. DNA concentration was determined using a NanoVue Plus spectrophotometer (GE Healthcare, Little Chalfont, Buckinghamshire, UK). The following VDR SNPs were selected based on a literature review: FokI (rs2228570), BsmI (rs1544410), ApaI (rs7975232), and TaqI (rs731236) [30,31]. SNP detection was achieved via polymerase chain reaction (PCR): Amplification of SNP regions was performed in a 25 μL reaction volume containing 50 ng genomic DNA, Milli-Q water, buffer, 3.0 mM MgCl_2_, 0.2 mM of each dNTP, 20 pM of each primer, and 1 U Taq DNA polymerase. The nucleotide sequences of each primer and the expected fragment sizes are as follows [30,31]:

TaqI (T/C) rs731236

Forward: 5′-CAGAGCATGGACAGGGAGCAA-3′
Reverse: 5′-GCAACTCCTCATGGCTGAGGTCTC-3′ (expected fragment size: 745 bp)

FokI (T/C) rs2228570

Forward: 5′-AGCTGGCCCTGGCACTGACTCTGGCTCTG-3′
Reverse: 5′-ATGGAAACACCTGCTTCTTCTCCCTC-3′ (expected fragment size: 265 bp)

BsmI (A/G) rs1544410

Forward: 5′-AGTGTGCAGGCGATTCGTAG-3′
Reverse: 5′-ATAGGCAGAACCATCTCTCAG-3′ (expected fragment size: 191 bp)

ApaI (T/G) rs7975232

Forward: 5′-GCAGAGCATGGACAGGGAGCAA-3′
Reverse: 5′-CAGGGAAGGAAGTAAGGGGAAGG-3′ (expected fragment size: 740 bp)

For the SNPs ApaI (rs7975232) and TaqI (rs731236), the PCR cycling conditions were as follows: initial denaturation at 95 °C for 5 min; 35 cycles of denaturation at 95 °C for 20 s, annealing at 59 °C for 30 s, and extension at 72 °C for 2 min; followed by a final extension at 72 °C for 10 min.

For FokI (rs2228570), the PCR conditions included initial denaturation at 95 °C for 5 min, followed by 35 cycles of denaturation at 95 °C for 45 s, annealing at 58 °C for 45 s, and extension at 72 °C for 45 s, with a final extension at 72 °C for 10 min.

For BsmI (rs1544410), the protocol consisted of initial denaturation at 94 °C for 4 min, 35 cycles of denaturation at 94 °C for 30 s, annealing at 58.5 °C for 30 s, and extension at 72 °C for 30 s, followed by a final extension at 72 °C for 5 min.

PCR products were separated by electrophoresis on 2% agarose gels stained with 0.1% ethidium bromide (4 μL in 50 mL agarose gel) and visualized under a UV transilluminator (UVP, Upland, CA, USA).

PCR product digestion—restriction fragment length polymorphism (RFLP) analysis:

Following amplification, 10 μL of each PCR product was digested with the appropriate restriction enzymes: FokI, ApaI, and BsmI (New England BioLabs, Boston, MA, USA) and TaqI (Jena Bioscience, Munich, Germany). These enzymes cleave the PCR products at specific recognition sites, resulting in fragments of varying lengths. Digestions were performed for 1 h at a constant temperature according to the manufacturers’ instructions [32].

Genotyping and allele analysis of the VDR SNPs were conducted at the Scientific Veterinary Institute of Serbia, Belgrade. Genotypes and alleles were determined based on the presence (lowercase letters) or absence (uppercase letters) of restriction sites.

### 2.3. Statistical Analysis

Data analysis was performed using IBM SPSS Statistics software, version 25.0 (IBM Corp., Armonk, NY, USA). Hardy–Weinberg equilibrium was assessed. Qualitative variables were compared using the chi-square test or Fisher’s exact test, depending on data distribution. For quantitative variables, the Student’s *t*-test and one-way analysis of variance (ANOVA) were applied when data were normally distributed; otherwise, the nonparametric Mann–Whitney U test was used. Associations between vitamin D levels and SNP genotypes were evaluated, with statistical significance set at *p* < 0.05.

## 3. Results

In this study of 60 patients with DES aged over 18 years and recruited from the Department of Ophthalmology at the University Clinical Center of Serbia in Belgrade, the relationship between VDR polymorphism genotypes and serum vitamin D [25(OH)D_3_] concentration was investigated. Patients were categorized according to their vitamin D status as follows:

- Deficient (<20 ng/mL).
- Insufficient (20–30 ng/mL).
- Sufficient (>30 ng/mL)

For each VDR polymorphism (TaqI, FokI, ApaI, BsmI), genotype distribution analysis was performed in relation to vitamin D status [Table 1].

### 3.1. Genotype Distribution and Hardy–Weinberg Equilibrium

For all analyzed polymorphisms, the frequency of the dominant alleles was pronounced, approximately 0.75 for TaqI, FokI, and ApaI, while slightly lower at 0.71 for the BsmI polymorphism. This distribution indicates a strong presence of dominant variants within the studied population. When expected values under Hardy–Weinberg equilibrium were compared with the observed genotype distributions, no significant imbalance was detected, suggesting that the population is likely in genetic equilibrium. Observed deviations—such as an increased number of homozygous dominant genotypes for FokI and ApaI—did not reach statistical significance but may point to subtle factors like genetic drift or the relatively small sample size [Table 2].

The overall association between genotypes and vitamin D levels was highly statistically significant (χ^2^ = 91.77; *p* < 0.0001). Among the individual SNPs, only the ApaI polymorphism (rs7975232) showed a statistically significant association with vitamin D levels (*p* = 0.0384). The other polymorphisms (TaqI, FokI, BsmI) did not exhibit significant associations within this population. Statistical significance for associations between vitamin D levels and SNPs was accepted at *p* < 0.05 [Table 3].

### 3.2. Genotypes and Vitamin D Status

When individual genotypes were analyzed in the context of vitamin D status, several notable distributions were observed:

TaqI (rs731236): The TT genotype was most prevalent among individuals with sufficient vitamin D levels, whereas the recessive genotype tt was found exclusively in patients with optimal vitamin D status. This distribution suggests that the t allele may be associated with better vitamin D status, although this was not confirmed as statistically significant.

FokI (rs2228570): The homozygous dominant genotype (FF) was most frequently observed among individuals with vitamin D deficiency. Although this polymorphism is hypothesized to affect the efficiency of vitamin D activation, no significant association with serum 25(OH)D_3_ concentrations was detected in this sample.

ApaI (rs7975232): This polymorphism was the only one to demonstrate a statistically significant association with vitamin D levels (*p* = 0.0384). The homozygous AA genotype was most frequently observed in individuals with vitamin D deficiency, whereas the heterozygous Aa genotype was more common among those with adequate vitamin D status. Conversely, the recessive aa genotype was present in patients with both deficient and insufficient vitamin D levels, as well as in some individuals with optimal levels, suggesting a potentially complex regulatory mechanism influenced by additional factors such as lifestyle habits and sun exposure.

BsmI (rs1544410): Genotype distribution did not reveal a statistically significant association with vitamin D concentration, although the BB genotype predominated in patients with deficiency. Heterozygous Bb and recessive bb genotypes were more frequently observed in individuals with low to borderline vitamin D levels, indicating a possible subtle trend that may have been obscured due to the limited sample size.

The overall analysis of the association between vitamin D status and the observed genotypes indicated a highly statistically significant relationship (χ^2^ = 91.77; *p* < 0.0001). This finding supports the presence of genetic determinants that may contribute to variations in vitamin D metabolism among patients with DES. However, when individual polymorphisms were analyzed separately, only ApaI (rs7975232) exhibited a significant association (*p* = 0.0384), while TaqI, FokI, and BsmI did not reach statistical significance (*p* > 0.05). This disparity may reflect the distinct biological functions of each SNP within the VDR gene, as well as their differential roles in modulating the transcriptional activity of the vitamin D receptor.

## 4. Discussion

Our results demonstrate that the majority of patients with DES exhibit low serum levels of 25-hydroxyvitamin D [25(OH)D_3_], with most individuals classified as deficient or insufficient. These findings corroborate previous studies that have identified vitamin D deficiency as a common feature among patients with various ocular and autoimmune disorders [33,34,35]. Vitamin D plays a pivotal role in immune regulation broadly, and more specifically, it has non-structural functions critical for maintaining epithelial barrier integrity and tear film stability. Its effects are mediated through the vitamin D receptor (VDR), which is expressed in multiple ocular tissues, including the cornea, conjunctiva, and lacrimal glands, indicating that local vitamin D activity may have direct relevance to the pathophysiology of DES [36,37,38]. Vitamin D deficiency has been linked to enhanced expression of proinflammatory interleukins such as IL-1β, IL-6, and TNF-α, which are physiological mediators of the inflammatory response and are frequently elevated in the tears of DES patients. Moreover, vitamin D is known to inhibit the differentiation of Th17 lymphocytes, which contribute to ocular surface damage in chronic inflammatory conditions. Within this context, low vitamin D concentrations may exacerbate symptom severity and disease progression, potentially facilitating worsening of clinical manifestations [39,40]. A major protective mechanism of vitamin D involves suppression of the NF-κB signaling pathway, thereby reducing inflammatory activation [41]. Monitoring vitamin D deficiency is particularly important in relation to meibomian gland dysfunction, as this directly impairs the lipid layer of the tear film and increases tear evaporation [42,43]. Vitamin D regulates the metabolism of antimicrobial peptides, such as cathelicidin, which play a crucial role in protecting the ocular surface. Reduced expression of these molecules in individuals with vitamin D deficiency may contribute to impaired immune barrier function and secondary infections, which are frequently associated with DES [42].

While it is well established that vitamin D status depends on factors such as UV exposure, diet, and age, increasing attention is being directed towards genetic determinants, particularly polymorphisms in the vitamin D receptor (VDR) gene [44]. In our study, we analyzed the four most commonly investigated SNPs: TaqI, FokI, ApaI, and BsmI. The most notable finding concerns the ApaI polymorphism (rs7975232), which demonstrated a statistically significant association with vitamin D status (*p* = 0.0384). The homozygous AA genotype was predominantly observed in patients with pronounced deficiency, whereas heterozygous (Aa) and recessive homozygous (aa) genotypes were more frequently associated with better nutritional vitamin D levels. Previous studies have suggested that although this polymorphism is intronic, it may influence the stability of VDR mRNA, resulting in reduced protein expression, diminished transcription of target genes, and less effective immune regulation [30,44].

Although the other SNPs investigated in our study (TaqI, FokI, BsmI) did not reach statistical significance in association with vitamin D levels, certain trends were observed that suggest that these variants might have clinical relevance in larger cohorts. For example, the TaqI tt genotype was exclusively found in patients with sufficient vitamin D levels, indicating a potential protective role; however, this trend lacked statistical confirmation due to the limited sample size.

As modern ophthalmology and therapeutic strategies increasingly move towards a personalized approach in the management of DES, the identification of VDR genotypes may hold significant clinical value. Genotyping can aid in recognizing patients predisposed to lower vitamin D levels who are consequently at a higher risk of developing or experiencing exacerbated DES symptoms. For these patients, regular monitoring of vitamin D status and early supplementation could become integral components of a targeted therapeutic protocol.

Given that treatment for DES is continually evolving and tailored according to individual clinical presentations, it is crucial to incorporate genotypic profiling into patient management. Current therapies primarily involve local interventions—such as artificial tears, anti-inflammatory agents, and immunomodulators—but accumulating evidence suggests that systemic vitamin D supplementation, especially in patients with documented deficiency, may improve both subjective symptoms and objective disease parameters [45,46]. Our study supports this concept and opens avenues for further research investigating therapeutic outcomes stratified by VDR genotype.

In conclusion, accumulating evidence suggests that VDR polymorphisms may influence receptor functionality by altering protein isoform structure, mRNA stability, and transcriptional efficiency. These molecular modifications can disrupt the regulation of cytokine expression and T-cell differentiation, thereby promoting ocular surface inflammation and contributing to the immunopathogenesis of DES.

Nevertheless, it is important to acknowledge certain limitations inherent to our study. Although the findings are meaningful, the relatively small sample size (n = 60) may limit statistical power and the ability to detect subtle associations. We implemented stringent inclusion and exclusion criteria to homogenize the study cohort and enhance result relevance, yet the sample size remains modest for a genetic association study. Future research should aim to increase participant numbers and broaden the geographical scope to validate and extend these findings.

Future studies should include larger and more demographically diverse populations, as well as longitudinal designs that would allow for the assessment of vitamin D therapy effects in patients with different VDR genotypes. It would also be valuable to investigate the local expression of VDRs in the conjunctiva and cornea, alongside cytokine profiles of the tear film, in relation to genotype and vitamin D nutritional status. Such a multidimensional approach could substantially enhance our understanding of the molecular mechanisms linking systemic deficiency to local inflammatory processes in the eye.

## 5. Conclusions

As seen in the results of our study, a strong association was observed between low serum levels of 25-hydroxyvitamin D [25(OH)D_3_] and the presence of DES, further reinforcing the hypothesis of vitamin D’s role in maintaining ocular surface homeostasis and integrity. Chronic vitamin D deficiency, as evidenced by our data, may contribute to tear film dysfunction and the development of the inflammatory response characteristic of DES, potentially acting as a factor that exacerbates disease symptoms or reduces the efficacy of conventional therapies.

Genetic analysis revealed a statistically significant correlation between the ApaI (rs7975232) polymorphism in the vitamin D receptor (VDR) gene and 25(OH)D_3_ status, with the AA genotype being more frequently observed in patients with vitamin D hypovitaminosis. This finding suggests that genetic variability within the VDR gene may influence the binding affinity and expression of vitamin D, which could have direct implications for immune regulation and the epithelial stability of the ocular surface.

These results highlight the potential utility of VDR polymorphism genotyping as an adjunct diagnostic tool, particularly in cases of chronic and treatment-resistant DES. Incorporating molecular markers into clinical practice would enable patient stratification based on individual risk profiles and facilitate personalized vitamin D supplementation strategies tailored to genetic predispositions. This approach is especially relevant given that standard DES therapies do not always yield satisfactory outcomes, whereas targeted correction of vitamin D deficiency, combined with pharmacological treatment, may improve clinical prognosis.

In summary, the findings of this study pave the way for novel diagnostic and therapeutic avenues in DES by linking nutritional and genetic factors, thereby underscoring the importance of integrating molecular genetic medicine into ophthalmological practice.

## Figures and Tables

**Table 1:** Genotype distribution and vitamin D status.

Polymorphism	Genotype	Deficient	Insufficient	Sufficient	Total	Allele
TaqI (rs731236)	TT (M)	4	4	25	33	T
	Tt (H)	1	2	16	19	t
	tt (W)	0	0	5	5	
**Total (n)**		5	6	46	57	
FokI (rs2228570)	FF (W)	22	7	5	34	F
	Ff (H)	6	5	5	16	f
	ff (M)	2	2	2	6	
**Total (n)**		30	14	12	56	
ApaI (rs7975232)	AA (M)	20	10	7	37	A
	Aa (H)	9	2	4	15	a
	aa (W)	0	5	2	7	
**Total (n)**		29	17	13	59	
BsmI (rs1544410)	BB (M)	16	7	7	30	B
	Bb (H)	15	2	2	19	b
	bb (W)	3	2	2	7	
**Total (n)**		34	11	11	57	

**Table 2:** Allele frequency analysis and Hardy–Weinberg equilibrium distribution results.

Polymorphism (rsID)	Observed Genotypes (n)	Allele Frequencies (*p*/*q*)	Expected HWE Distribution (Hom Dom/Het/Hom Rec)	χ^2^ (HWE)	*p*-Value
TaqI (rs731236)	TT: 33, Tt: 19, tt: 5	*p* = 0.75, *q* = 0.25	31.7/21.6/3.7	0.52	0.47
FokI (rs2228570)	FF: 34, Ff: 16, ff: 6	*p* = 0.75, *q* = 0.25	31.5/21.0/3.5	1.10	0.29
ApaI (rs7975232)	AA: 37, Aa: 15, aa: 7	*p* = 0.75, *q* = 0.25	33.6/21.9/3.6	1.65	0.20
BsmI (rs1544410)	BB: 30, Bb: 19, bb: 7	*p* = 0.71, *q* = 0.29	27.9/23.3/4.9	0.87	0.35

Note: All SNPs conformed to Hardy–Weinberg equilibrium (*p* > 0.05).

**Table 3:** Assessment of the association between vitamin D levels and SNPs, showing statistical significance.

Polymorphism (rsID)	χ^2^	df	*p*-Value
TaqI (rs731236)	2.35	2	0.125
FokI (rs2228570)	1.89	2	0.169
ApaI (rs7975232)	6.53	2	0.0384 *
BsmI (rs1544410)	0.98	2	0.322
Overall	91.77	-	<0.0001 ***

Note: *p* < 0.05 was considered statistically significant. * Statistically significant. *** Highly significant.

## Data Availability

The raw data supporting the conclusions of this article will be made available by the authors on request.
